# Supplementary material for: Towards artificial intelligence-based disease prediction algorithms that comprehensively leverage and continuously learn from real-world clinical tabular data systems
Source: PLOS Digit Health. 2024 Sep 3;3(9):e0000589. doi: 10.1371/journal.pdig.0000589 (PMC11371204; doi:10.1371/journal.pdig.0000589)
Supplement: Attachment — Submitted filename: Response to Reviewers.docx [file pdig.0000589.s002.docx]

**Reviewer #1**

**This paper addressed a common problem faced by physicians and presented a classic case of how AI may help clinical practice. However, the technical depth needs to be strengthened. There should be more in-depth discussion on the structure/type of the neural network used, what was the parameter set up, what was the considerations, etc. Otherwise it'll be simply another case of using an off the shelf model on some data.**

We have added a new figure outlining the full algorithm – see the section titled **Summary of Full Algorithm**, Figure 2. Here we provide a visual that details the neural network architecture we utilize (previously only provided in text), as well as summarizes other aspects of the full algorithm that contribute to automating the production of a disease prediction model. However, while we agree that adding this strengthens the manuscript, we disagree with Reviewer 1’s rationale for this suggestion – namely, that without it, our approach would be comparable to an “off the shelf model.”

We do not claim to utilize a novel neural network architecture or other technically complex methods, and so the uniqueness of our approach is not contingent on understanding underlying complexities. Instead, our work is simply an application of a series of simple steps (see Figure 2.) which combine to create a risk calculator that is able to learn from a live, real-world tabular data system. The simplicity of our approach is discussed throughout the paper, and is even credited as a major reason why our approach is feasibly to undertake locally (see **NN Training** and **General Advantages of this Strategy/Algorithm** sections).

We believe it is important to recognize that our goal itself is unique because we advocate for conceptualizing risk estimation as a local endeavour. Our aim, to create a strategy that is feasibly to apply locally to real-world data systems, is what sets our work apart from traditional projects that typically aim to create a specific risk prediction model based on a specific dataset.

Traditionally, when an off-the-shelf risk calculator is developed and presented as high performing, the implied goal is “broad application” of the specific prediction model – a developer will use a sample dataset, construct features, train a risk prediction model, show model performance, then advocate for its broader adoption (or at least imply such adoption is warranted) when estimating risk.

We, on the other hand, do not suggest that a specific prediction model is the end-product of our work. Instead, we sought out to create a generalizable strategy that can automate the creation of a unique risk calculator that reflects local and contemporary data patterns, and therefor can more reasonable be expected to appropriately apply to local patients. Additionally, our goal was also to create a strategy that automates the comprehensive utilization of all available tabular patient data so that local data sources are fully leveraged when estimating risk in the proposed manner.

Thus, our strategy, while comprised of simple steps, stands in stark contrast to traditional off-the-shelf models, which (1) are typically developed using a static dataset from a specific location or point in time, and (2) explicitly incorporate a fixed set of predictor variables based on content expertise. We acknowledge that it is certainly feasible for an off-the-shelf risk model to be “refined” locally (if first pre-trained on an original dataset, then trained again on local data) to enhance local generalization. However, this option is not typically offered as a standard feature to our knowledge. Additionally, even if offered, we have never encountered an off-the-shelf risk model that utilizes automated predictor selection (occurring external from the model training) as a mechanism for model refinement. We therefor believe that our approach is fundamentally unique despite the lack of technical complexities in the methods we employ.

**In addition, the clinical relevance of the result should be included.**

Disease risk calculators provide clinically relevant information by accurately identifying patients at risk for future disease. Predictions from such models have established clinical utility, as they are already commonly used for preventative purposes globally. Typically, development of traditional risk calculators is based on well-known risk/protective factors, and does not itself provide unique scientific contributions to the underlying clinical theory of disease. In a similar vein, we are not purporting that our proposed algorithm produces predictive models that are interpretable in a manner that contributes to clinical theory. We are only assuming what is standard in this field – that an accurate prediction itself has clinical utility for preventative purposes.

Because the strategy proposed in this manuscript is only clinically relevant if it produces risk models that accurately identify patients in a real-world setting, we have focused on reporting predictive performance metrics (i.e., AUROC, AUPRC, PPV, sample N’s and incidence) calculated from our real-world PoC and contextualizing these results. We are unclear as to what additional information (to demonstrate clinical utility) this comment is asking for beyond these data.

**Reviewer #2**

**The manuscript presents a proof-of-concept for an algorithm that could revolutionize disease risk estimation using real-world clinical data. By ingeniously combining classic statistical methods with cutting-edge artificial intelligence techniques, the researchers have developed a versatile algorithm capable of autonomously navigating through complex clinical data systems like electronic health records (EHR) or claims databases. What sets this algorithm apart is its self-adaptive nature, constantly evolving to reflect changes within the data system, thus ensuring the accuracy and relevance of risk estimates over time.**

**The comprehensive approach of the algorithm, which considers a multitude of factors including encounters, diagnoses, procedures, medications, labs, and demographics, without the need for extensive data curation, is a remarkable departure from traditional static prediction models. The authors' rigorous validation process, spanning a significant timeframe and involving a large patient cohort, demonstrates the algorithm's robustness and effectiveness in estimating the risk of critical events like stroke or myocardial infarction.**

**The reported AUROC values and odds ratios provide compelling evidence of the algorithm's performance and its potential to significantly enhance clinical decision-making. Moreover, the observed improving trend over time underscores the algorithm's capability to continuously learn and adapt, promising even greater accuracy and reliability in the future.**

**In summary, this manuscript represents a major advancement in the field of predictive analytics in healthcare. The algorithm's ability to harness the full spectrum of information within real-world clinical data systems, coupled with its adaptive nature and impressive performance metrics, paves the way for the development of highly effective disease risk calculators that can truly revolutionize patient care.**

Thank you for your positive review.

We believe that our strategy, though not overly complex, is paradigm shifting and will hopefully lead to increased local development of risk calculators, as such calculators are likely to reflect health dynamics most relevant to local populations.

We would also like to point out that given the flexible nature of our proposed strategy, if the underlying data system incorporates new types of tabular data (e.g., nomics, health monitors, structured data retrieved from physician notes using LLMs, volumetric data drawn from imagine studies, etc), such data can readily be folded into the full algorithm so that updated risk estimates can potentially reflect these new data types as well.

Ultimately, the strategy described in the manuscript attempts to leverage all informative tabular data that are available in the local system, and so in theory will be able to improve prediction as tabular data systems become increasingly comprehensive in the future.

**Reviewer #3**

**In my opinion, although the faced problem is interesting, the work needs further improvements in order to reach a version to publish and that could effictively used by the sceintific community.**

**First, It's unclear how the integration of social behavior factors with environmental variables contributes to more accurate predictions.**

We do not believe this comment is relevant to our submission because we do not discuss social behavioral factors or environmental variables anywhere within our manuscript.

**I suggest proofreading since the paper is affected by several typos and grammatical errors.**

We have made edits to the text throughout in attempt to improve grammar, clarity, and readability.

**Furthermore, the lack of a precise and detailed discussion about the direction of the article, and specifically a clear explanation of motivation, scenario and overall contribution make the paper not easier to follow and understand.**

This comment is a bit confusing to us because we feel that we were very clear on these points. As is stated in the introduction, the point of the article is to present a generalizable strategy that (when applied to a real-world clinical data system) can automate the creation of disease risk calculators. Our motivation is also stated in the introduction: (1) that when applied in a real-world setting, traditional calculators often do not generalize well to patients from distant context/locations, and (2) that traditional calculators do not comprehensively utilize all available clinical data, but instead base the feature selection on well-known risk/protective factors. Thus, our contribution (which is also discussed in the introduction) is that we present a strategy that aims to improve predictive performance by more comprehensively leveraging available clinical data and to overcome traditional issues related to lack of generalizability by formulating a strategy that can be implemented on any local real-world data system.

**Second, the proposed method appears overly complex with the integration of multiple neural network architectures like LSTM, gate recurrent unit, BiLSTM, and attention mechanisms. This complexity might hinder the method's practicality and interpretability. This could help the reader to understand the novelty introduced by the paper. Actually, they not evident the advantages of the proposed method in comparison with the existing works.**

This comment is also confusing to us because despite what this reviewer claims, we did not integrate any of the complex neural network architectures named here. To the contrary, we only utilized a simple feed-forward architecture – this was stated clearly in the **Neural Network (NN) Structure** section. We believe this comment either reflects a major misreading of our manuscript, or perhaps is erroneously referring to a different submission.

**While the abstract mentions experimental results, it doesn't provide insights into how the proposed method performs in real-world scenarios. Without validation against real-world data, the practical relevance and generalizability of the method remain questionable.**

This comment is also confusing to us because despite what this reviewer claims, we do not mention experimental results in the abstract. Additionally, we clearly discuss real-world application of our proposed strategy, as that is intimately related to the motivation behind our project. Moreover, we do provide a PoC using a real-world EHR system, so the claim that there is no validation against real-world data is categorically incorrect. As with the comment above, we believe this comment also either reflects a major misreading of our manuscript, or perhaps is erroneously referring to a different submission.

**Due to the reasons given above, this paper should be rejected.**

For reasons discussed above, we feel that reviewer #3’s comments overall do not reflect a careful reading of our manuscript and/or are not relevant to our submission.
